# Effect of different surgical methods on headache associated with cervical spondylotic myelopathy and/or radiculopathy

**DOI:** 10.1186/s12893-015-0092-3

**Published:** 2015-09-23

**Authors:** Yuqing Sun, Aikeremujiang. Muheremu, Kai Yan, Jie Yu, Shan Zheng, Wei Tian

**Affiliations:** Department of Spine Surgery, Beijing Ji Shui Tan Hospital, 31 Xinjiekou East Street, Xicheng District, Beijing 100035 China; Medical Center, Tsinghua University, Haidian District, Beijing 8610084 China

**Keywords:** Anterior cervical discectomy and fusion, Total disk replacement, Open door laminoplasty, Headache, Cervical spondylosis

## Abstract

**Background:**

Anterior cervical discectomy and fusion, total disk replacement and open door laminoplasty have been widely used to treat patients with cervical spondylotic myelopathy and/or radiculopathy. In our clinical practice, many patients with cervical spondylosis also complain of headache, and wish to know if the surgical treatment for cervical spondylosis can also alleviate this symptom. Considering that there is no literature concerning this extra benefit of surgical manipulation on cervical spondylosis, we have carried out this retrospective study.

**Methods:**

Among the patients treated with anterior cervical discectomy and fusion, total disk replacement and open door laminoplasty in our institute for cervical spondylotic myelopathy and/or radiculopathy between February 2002 to March 2011, 108 of whom that have complained about headache at the same time were included in this study. Those patients were followed by 25 to 145 months. Severity of headache before the surgery and at the last follow up was recorded by VAS pain scores and compared among the patients with different surgical methods using SPSS17.0 software. One way ANOVA was used to compare VAS scores between the groups, paired sample t-tests were used to compare the differences in a group at different time points.

**Results:**

Headache was significantly alleviated in all groups (P < 0.01). Respectively, 75.0 % of the patients in the ACDF group, 84.6 % of the patients in the TDR group and 82.2 % of the patients in the laminoplasty group were significantly relieved of the headache after the surgery. No significant differences were found with the VAS score at the last follow up among the groups (P > 0.05). No significant differences were found among the groups comparing the degree of alleviation of VAS scores before and after the surgery (P > 0.05).

**Discussion:**

Considering that all the three procedures in the current study have achieved similar effect on alliviating headache in patients with cevical myelopathy, and that what they have in common was that was the decompression of spinal cord, it can be assumed that the headache associated with cervical spondylosis may be the result of compression on the spinal cord.

**Conclusions:**

Anterior cervical discectomy and fusion, total disk replacement and open door laminoplasty can all significantly alleviate headache in patients with cervical spondylotic myelopathy and/or radiculopathy. No surgical technique is better than any other technique on alleviating cervical headache associated with cervical spondylotic myelopathy and/or radiculopathy.

## Background

Cervical spondylosis is one of the most common reasons for spinal cord or nerve root dysfunction among the people over 55 years of age [1, 2]. There are reports on the effectiveness of anterior cervical discectomy and fusion (ACDF) and total disk replacement (TDR) and corpectomy for patients with cervical spondylotic myelopathy and/or radiculopathy [3, 4]. ACDF and TDR can decompress the spinal cord and nerve roots, they were widely applied for the treatment of degenerative cervical disk diseases [5, 6]. Bilateral open-door expansive laminoplasty have been proven to be effective in alleviating symptoms of cervical spondylosis [7, 8]. Laminoplasty can decompress the spinal cord by widening the spinal canal. In the meanwhile, it preserves the posterior spinal structure, which helps preserving the stability of the spine and preventing kyphosis.

In our clinical practice, patients with cervical spondylosis often complain about headache, and always wish to know if the surgical treatment for cervical spondylosis can also alleviate this symptom. However, to our knowledge, few studies have reported the efficacy of any surgical approaches on headache associated with cervical spondylosis. In the current study, we have retrospectively compared the efficacy of anterior cervical discectomy and fusion, total disk replacement and open door laminoplasty on headache associated with cervical spondylotic myelopathy and/or radiculopathy, in order to provide some clues or evidence to further study the mechanism of headache and choose appropriate treatment method in this condition.

## Methods

### Patients

The clinical materials of the patients who received surgical intervention in the Beijing Jishuitan Hospital for the treatment of cervical spondylotic myelopathy and/or radiculopathy, and reported headache before the surgery were included in the study and were retrospectively analyzed. The study was approved by the ethical committee of Beijing Jishuitan Hospital. Patients provided written informed consent for the publication of their individual clinical details, and the procedures were in compliance with Helsinki Declaration.

### Inclusion criteria

We have treated 336 patients from Feb 2002 to Mar 2011 with ACDF, TDR and laminoplasty. Among them there are there were 108 patients (32.1 %) (20 in the ACDF group, 26 in the TDR group, 62 in the laminoplasty group) who has reported headache before the surgery. Those patients diagnosed with cervical spondylotic myelopathy and/or radiculopathy who also complained of headache and surgically treated in our department from February 2002 to March 2011 were included in the current study. The surgical methods include ACDF, TDR and laminoplasty based on the surgical indication criteria in Table 1.

**Table 1:** Inclusion criteria of patients to groups with different surgical methods

Methods	Inclusion criteria
ACDF	1. One or two level of cervical spinal cord compression is shown by the magnetic resonance imaging
	2. No abnormalities were reported by other specialists from neurology, ophthalmology, cardiovascular and otolaryngology.
	3. No improvement of symptoms was achieved after at least 3 months of conservative treatment.
	4. Have no history of cervical surgery.
	5. Patient chose ACDF over TDR.
TDR	1. One or two level of cervical spinal cord compression is shown by the MRI
	2. Myelopathy or refractory radiculopathy due to degenerative disc disease confirmed by magnetic resonance imaging and/or computed tomography myelogram
	3. No abnormalities were reported by other specialists from neurology, ophthalmology, cardiovascular and otolaryngology.
	4. No obvious facet joint arthrosis and no instability was found in the cervical spine.
	5. Have no history of cervical surgery
	6. Patient chose TDR over ACDF
Laminoplasty	1. More than two level of cervical spinal cord compression is shown by the MRI
	2. Abnormalities of neurology, ophthalmology, cardiovascular, otolaryngology were excluded
	3. No improvement of the symptom after at least 3 months of conservative treatment.
	4. Have no history of cervical surgery.
	5.Patient agreed to receive open door laminoplasty

### Surgical procedure

Twenty patients (Table 1) underwent anterior cervical discectomy and fusion. Twenty-six patients (Table 2) underwent TDR. TDR was performed through a standard Smith-Robinson approach according to the operation manual. After decompression (posterior longitudinal ligament routinely removed), the artificial disc was inserted. The Bryan TM disc (Medtronic, Minneapolis, Minnesota, USA) was used in 25 patients, and the Discover TM disc (Johnson& Johnson, NJ, USA) was used in one patient. Open door laminoplasty was performed in 62 patients (Table 3). All the patients underwent bilateral open-door expansive laminoplasty by sagittal splitting of the spinous process. The door was kept open by inserting artificial bone between the spinous processes.

**Table 2:** Demographic characteristics of the patients involved in the ACDF surgery group

Case	Sex	Age	Type	Follow up	Site	VAS	VAS	VAS
						BS	AS	BS-AS
1	M	48	1	145	C4/5,C5/6	3	3	0
2	M	59	1	138	C3/4,C4/5	2	0	2
3	M	48	2	127	C6/7	3	0	3
4	F	51	3	122	C4/5,C5/6	5	0	5
5	F	65	2	94	C6/7	3	1	2
6	F	48	3	103	C5/6	4	4	0
7	M	38	1	71	C4/5,C5/6	2	0	2
8	M	58	1	63	C4/5	3	0	3
9	F	49	1	57	C3/4	4	0	4
10	F	44	1	56	C4/5,C5/6	7	0	7
11	M	41	2	50	C6/7	9	3	6
12	M	44	1	48	C5/6	3	3	0
13	F	60	3	57	C3/4,C4/5	6	6	0
14	M	71	1	37	C3/4	4	4	0
15	F	63	1	47	C5/6	7	0	7
16	F	57	3	44	C5/6	8	0	8
17	F	63	1	42	C4/5	3	0	3
18	F	47	1	40	C5/6,C6/7	3	0	3
19	M	35	1	39	C4/5,C5/6	1	0	1
20	M	55	1	37	C5/6	9	5	4

Type 1: myelopathy; type2: radiculopathy; type3: radiculomyelopathy

*BS*: before the surgery, *AS*: after surgery

**Table 3:** Demographic characteristics of the patients involved in the TDR surgery group

Case	Sex	Age	Type	Follow up	Site	VAS	VAS	VAS
						BS	AS	BS-AS
1	F	63	1	88	C56	7	0	7
2	F	48	1	85	C56	6	2	4
3	F	52	1	80	C56	7	0	7
4	F	44	1	74	C56	10	0	10
5	M	63	3	72	C34,C67	5	3	2
6	M	45	1	72	C67	6	6	0
7	M	47	1	70	C56	9	0	9
8	M	70	1	62	C56,C67	3	0	3
9	M	58	1	60	C34	3	3	0
10	M	51	1	60	C45	6	2	4
11	M	42	2	53	C56,C67	3	0	3
12	F	39	1	51	C56	9	9	0
13	F	41	3	48	C45	7	5	2
14	M	38	1	48	C56	3	0	3
15	F	55	1	48	C45	9	4	5
16	F	45	3	48	C67	7	1	6
17	F	67	1	45	C56	5	0	5
18	M	43	2	45	C67	5	0	5
19	F	49	1	42	C56	4	3	1
20	M	31	1	42	C56	8	3	5
21	M	44	2	38	C45	9	3	6
22	M	52	3	38	C56,C67	7	0	7
23	F	38	3	37	C56	6	0	6
24	M	54	3	34	C67	0	8	−8
25	F	52	1	28	C56	7	4	3
26	F	50	3	22	C56	8	4	4

Type 1: myelopathy; type2: radiculopathy; type3: radiculomyelopathy

*BS*: before the surgery, *AS*: after surgery

### Data collection

All patients finished a questionnaire before the surgery and at the end of the follow up of 25–145 months. In the questionnaire, patients were asked to state the severity of headache by a scale of 0 to 10, with 0 having no headache and 10 as having headache in an unbearable level.

### Statistics

After the follow up, the VAS scores were compared using SPSS17.0 (Illinoi, USA). VAS score before the surgery and at the last follow up in the same group was compared by paired-sample t-tests and VAS score at different time points among the different groups was compared by one-way ANOVA. The calculated mean VAS scores were recorded as Mean ± Standard Deviation.

## Results

Twenty patients (10 male and 10 female) were treated with ACDF (Table 2). The mean age of those patients was 52.2 ± 9.7 (range 35–71 years). 26 patients (12 male and 14 female) were treated with TDR (Table 3). The mean age of those patients was 49.3 ± 9.4 (range 31–70 years). 62 patients (45 male and 17 female) were treated with open door laminoplasty (Table 4). The mean age of those patients was 54.2 ± 8.0 (range 39–73 years).

**Table 4:** Demographic characteristics of the patients involved in the laminoplasty surgery group

Case	Sex	Age	Type	Follow uptime	VAS	VAS	VAS
					BS	AS	BS-AS
1	M	52	1	114	3	0	3
2	M	56	1	124	3	0	3
3	M	64	1	122	3	0	3
4	M	54	1	121	2	0	2
5	M	50	2	116	2	0	2
6	M	52	3	115	5	0	5
7	M	62	1	115	3	0	3
8	M	43	1	115	5	3	2
9	F	48	1	112	2	0	2
10	M	53	1	107	4	0	4
11	M	60	1	76	3	4	−1
12	M	50	1	87	3	0	3
13	F	55	1	83	4	4	0
14	M	59	3	77	7	7	0
15	M	50	1	72	6	0	6
16	F	45	2	70	7	5	2
17	M	45	1	69	5	0	5
18	M	62	1	67	8	5	3
19	M	51	1	66	3	3	0
20	M	66	1	65	4	2	2
21	M	45	1	64	9	0	9
22	M	65	1	61	5	0	5
23	M	57	1	59	5	0	5
24	M	61	1	58	5	5	0
25	M	67	1	54	5	3	2
26	M	54	2	49	3	3	0
27	M	53	1	46	6	0	6
28	M	50	1	45	4	0	4
29	M	50	1	45	4	3	1
30	M	61	1	38	6	0	6
31	M	68	1	44	5	0	5
32	M	48	1	44	6	0	6
33	F	58	1	43	5	3	2
34	M	63	1	42	6	2	4
35	M	47	2	41	5	2	3
36	M	56	1	40	6	0	6
37	F	57	1	40	8	2	6
38	F	58	1	39	5	5	0
39	M	57	1	38	5	0	5
40	M	62	1	38	4	0	4
41	M	61	1	38	4	4	0
42	M	44	1	38	6	3	3
43	F	45	1	37	5	5	0
44	M	46	1	37	4	0	4
45	F	61	1	36	4	6	−2
46	F	41	1	34	9	4	5
47	M	45	1	33	8	0	8
48	M	40	1	32	9	6	3
49	M	53	1	32	7	4	3
50	F	48	3	31	8	0	8
51	F	67	1	30	4	0	4
52	M	57	1	30	3	3	0
53	F	65	3	30	2	0	2
54	M	59	1	29	4	2	2
55	F	73	1	27	4	2	2
56	M	52	1	27	1	0	1
57	F	39	1	26	3	3	0
58	F	49	1	27	6	3	3
59	M	41	1	27	9	0	9
60	F	52	1	26	5	0	5
61	F	47	1	26	7	3	4
62	M	64	1	25	6	3	3

Type 1: myelopathy; type2: radiculopathy; type3: radiculomyelopathy

*BS*: before the surgery, *AS*: after surgery

### Comparison of VAS scores at different time points in the same group

In the ACDF group (Table 2, Table 5), 15 patients out of 20 reported significantly alleviated symptoms, 12 of who reported that the headache completely disappeared after the surgery. The other 5 patients reported that there were no differences on the severity of headache after the surgery. The VAS score after the surgery was 1.5 ± 2.0, which was significantly (P < 0.001) lower than before the surgery (4.5 ± 2.4).

**Table 5:** VAS score of headache before the surgery and at the last follow up

Methods	N	Before	After	Difference	*P* value
ACDF	20	4.5 ± 2.4	1.5 ± 2.0	3.0 ± 2.6	<0.001
TDR	26	6.1 ± 2.4	2.3 ± 2.6	3.8 ± 3.6	<0.001
Laminoplasty	62	5.0 ± 1.9	1.8 ± 2.1	3.1 ± 2.4	<0.001
Total	108	5.1 ± 2.2	1.9 ± 2.2	3.3 ± 2.8	<0.001
P value		0.022	0.405	0.525	

Mean ± Standard deviation

In the TDR group (Table 3, Table 5), 22 patients out of 26 reported significantly alleviated symptoms, 11 of who reported that the headache completely disappeared after the surgery. 3 patients reported that there were no differences on the severity of headache after the surgery and one patient reported more severe headache at the last follow up than before the surgery. The VAS score at the last follow up was 2.3 ± 2.6, which was significantly (P < 0.001) lower than before the surgery (6.1 ± 2.4).

In the laminoplasty group (Table 4, Table 5), 51 patients out of 62 reported significantly alleviated symptoms, 31 of who reported that the headache completely disappeared after the surgery. 9 patients reported that there were no differences on the severity of headache after the surgery and 2 patients reported more severe headache at the last follow up than before the surgery. The VAS score at the last follow up was 1.8 ± 2.1, which was significantly (P < 0.001) lower than before the surgery (5.0 ± 1.9).

### Comparison of VAS scores between the groups

Before the surgery, VAS in the TDR group was significantly higher than the ACDF group (mean difference 1.7 ± 0.6, P = 0.010) and the laminoplasty group: (mean difference 1.2 ± 0.5, P = 0.022). There were no statistical differences among the VAS scores of different surgery groups after the surgery (P > 0.05). No differences were found among the groups comparing the degree of improvement (VAS _before the surgery - after the surgery_) of VAS scores after the surgery (Table 5).

### Comparison of VAS scores between different levels of cervical pathology

To find if the incidence of headache was related to the level of radiologically significant cervical pathology, we respectively analyzed the number of patients with cervical pathology at the level of C3/4, C4/5, C5/6, C6/7 among patients who received ACDF and TDR, and the number of patients with headache among the patients with cervical pathology at each level. As a result, the incidence of headache in patient with significant pathological change at C3/4, C4/5, C5/6, C6/7 are 30 %, 28.3 %, 30.3 %, 46.2 % (Table 6). No significant difference was found by chi square tests regarding the incidence of headache among patient with pathology at different cervical level (P > 0.05).

**Table 6:** Total number of patients, and the number of patients with headache with the radiologically confirmed cervical pathology, and the value of VAS reduction after the surgical intervention

	C_3/4_	C_4/5_	C_5/6_	C_6/7_
Total	20	46	89	26
Headache	6	13	27	12
VAS change	1.3 ± 1.6	3.1 ± 2.2	4.2 ± 2.9	2.7 ± 4.0

We have also compared the degree VAS score reduction among the different levels of cervical pathology with one way ANOVA method. As a result, patients underwent surgery at the level of C6/7 achieved significantly more alleviation of headache than C3/4 (P = 0.033). No significant differences were found among other levels (Table 6).

## Discussion

Cervical spondylosis is the most common reason of spinal cord related abnormalities among people over 55 [9, 10]. In the elderly population, degeneration and protrusion of cervical intervertebral disk causes compression of spinal cord and nerve roots, which impairs sensory and motor functions of the spinal cord and peripheral nerves. Numbness, hypersensitivity and pain on the neck and shoulder, impairment of the fine-motor performance of arms, difficulty in fast movement and abnormal reflexes of extremities are often found in patients with cervical spondylosis. In severe cases, trouble in walking steadily, active reflex of tendons and atrophy of muscles of the extremities can be observed [11–14].

In our clinical practice, except from the above mentioned common manifestations, many patients with cervical spondylosis also complain about headache. They always wish to know if the surgical treatment for cervical spondylosis can also effectively alleviate their headache to a level that does not affect their daily lives. Although we find that some of the patients did experience significantly alleviated headache after the surgery, we find no literature that have provided evidence on the effectiveness of surgical manipulation on the alleviation of headache in patients with cervical spondylosis. Thus we have retrospectively reviewed the patients complaining of headache and received surgical treatment in our department to find out whether the surgical manipulation can have a positive effect on headache

In the current study, most patients achieved significant alleviation of headache after the surgery. Among the 108 patients, 88 patients reported significantly alleviated headache, 64 of who reported that the headache completely disappeared after the surgery. Only 3 patients, one in the TDR group and two in the laminoplasty group, reported worsened headache after the surgery.

Comparing the efficacy of different surgical methods, no significant difference was found among the groups on the severity of headache at the last follow up, and no differences were found comparing the reduction of VAS score after the surgery among groups. The reason why the VAS score was significantly higher in the laminoplasty group compared to other groups may be because patients in this group usually suffered from three or more level of cervical spinal cord compression while patients in the other groups suffered from less than two levels of cervical spinal cord compression. Considering that the difference in the VAS score before the surgery may affect the meaning of the VAS scores at the last follow up, we have also compared the changes in VAS score among the groups in addition to the VAS scores at the last follow up, and both results indicate that no surgical method is significantly better than any other method in alleviating the severity of headache in patients with cervical spondylosis. The current study has also sought to find a relationship between the level of cervical pathology and headache, but significant differences were found among the incidence of headache and the location of cervical pathology. Although patients with cervical pathology at the level of C5/6 seem to achieve significantly more relief than patients with C3/4 cervical pathology, since there were only 6 patients with C3/4 pathology, it may still not be adequate to say that lower cervical pathology had better pain relief than upper cervical lesions.

Although the current study showed the effectiveness of several types of surgical interventions on alleviating headache on patients with cervical spondylosis, as there is no radiological or pathological studies on the etiology of headache associated with cervical spondylosis in the current literature, we still don’t know for sure the mechanism of headache in these patients and why the surgical intervention for cervical spondylosis can significantly alleviate it and why there are no differences among the different surgical options on their ability to alleviate headache in patients with cervical spondylosis.

It is possible that mechanical oppression of uncovertebral osteophytes on vertebral artery and the sympathetic nerves on the vertebral artery could affect the blood supply to the brain, causing a set of symptoms including headache. However, in the current study, the preoperative MRI studies did not reveal such pathological change, and the surgical manipulation did not involve any procedures near the cervical vertebra but still achieved significant relieving of headache in most patients. This proves that compression of vertebral artery is not likely the cause of headache associated with cervical spondylosis. Some authors proposed that the headache associated with cervical spondylosis may be the result of the stimulation of sympathetic nerves in the posterior longitudinal ligament [15]. The fact that a network of sympathetic nerves were found in the posterior longitudinal ligament in several cadaveric and animal studies [16–19], and that relieving the compression of herniated intervertebral disks on the posterior longitudinal ligament or removing part of the posterior longitudinal ligament have effectively alleviate headache in patients from ACDF [20] and TDR group in the current study are in accordance with this hypothesis. However, in our study, no significant differences were found among the groups comparing the degree of alleviation of VAS scores before and after the surgery (P > 0.05). Considering that no herniated intervertebral disks were taken out, and that the posterior longitudinal ligament is intact during the laminoplasty, and that what all the three procedures in our study have in common is that direct compression on the spinal cord was relieved by the surgery, we believe that the headache associated with cervical spondylosis may not be the result of stimulation of the sympathetic nerves in posterior longitudinal ligament, but rather the compression of spinal cord itself. Further research should be carried out to explore the underlying mechanism of this symptom.

## Conclusions

Anterior cervical discectomy and fusion, total disk replacement and open door laminoplasty can all significantly alleviate headache in patients with cervical spondylotic myelopathy and/or radiculopathy. No surgical technique is better than any other technique on alleviating cervical headache associated with cervical spondylosis.
